# Adsorption Behaviors of 17*α*-Ethinylestradiol in Sediment-Water System in Northern Taihu Lake, China

**DOI:** 10.1155/2014/371075

**Published:** 2014-07-24

**Authors:** Yonghua Wang, Liangfeng Hu, Qiuying Wang, Guanghua Lu, Yi Li

**Affiliations:** Key Laboratory of Integrated Regulation and Resource Development on Shallow Lakes of Ministry of Education, College of Environment, Hohai University, Nanjing 210098, China

## Abstract

Adsorption behavior of 17*α*-ethinylestradiol (EE2) in northern Taihu Lake sediment was analyzed by using batch equilibrium experiment. Freundlich isotherm could describe the adsorption thermodynamic behavior of EE2 in sediment. Sediment organic matter (SOM) contents had important impacts on the adsorption capacity for EE2. The pH values also influenced the adsorption capacity for EE2. Increase of pH value could decrease the EE2 adsorption, which might be due to the electrostatic repulsion between the anionic form of EE2 and sediments with negative charge under high pH values. Competitive effects of bisphenol A (BPA) on EE2 adsorption were further analyzed. The results showed that low concentration BPA did not have significant influences on EE2 adsorption. However, high concentration BPA could reduce EE2 adsorption, which might be due to the similar molecular diameter of BPA with adsorption sites and one more benzene ring with a hydroxyl group in BPA. These results provide primary information of EE2 adsorption in sediment-water system in Taihu Lake, which is useful for the environmental risk assessment and management of EE2 in studied area.

## 1. Introduction

The 17*α*-ethinylestradiol (EE2), a kind of synthetic estrogen, is mainly used to treat the ailments of infertility, hormone imbalance, osteoporosis, and so on [1]. EE2 can exhibit strong biological effects at very low concentration, and its effect is about 10 times the natural estrogens. Thus, it is considered as one of the most active environmental endocrine disruptors (EDCs) [2, 3]. EE2 is relatively stable in the environment. Among its researches, the adsorption process of EE2 in the sediment-water system is an important field, since the adsorption of EE2 influences its fate, bioavailability, and persistence in the aquatic environment. The information can provide important support for environmental assessment and management of EE2.

Taihu Lake, the third largest freshwater lake of China, is located in the southeast of Yangtze River Delta, with an average depth of 1.9 m and surface water area of ~2338 km^2^ [4]. Taihu Lake not only provides agricultural and industrial water for surrounding cities such as Shanghai, Suzhou, and Wuxi, but also provides the drinking water source for several cities. Thus, the water quality of Taihu Lake has important influences on the residents' health in the basin. However, due to the growth of population and the development of industry and agriculture, some pollutants have been exhausted into Taihu Lake, which cause great environment pressure for ecosystem and human [5]. EDCs including EE2 are one kind of pollutants found in northern Taihu Lake. In order to effectively assess their ecological and health risk, it is necessary to identify their adsorption behavior and fate in sediment-water system. However, no available researches in northern Taihu Lake are found.

In this study, we investigated the adsorption behavior of EE2 in sediment-water system of northern Taihu Lake. After the analysis of kinetics and isotherms, the influences of sediment organic matter (SOM) and pH on EE2 adsorption were characterized. Since bisphenol A (BPA) as another important EDC has been widely detected in northern Taihu sediment, and competitive adsorption of EE2 and BPA in sediment and the selectivity of the sediment were not clear, the influences of BPA on EE2 adsorption were also analyzed in this study. This study provided primary information for further study of migration, conversion, and other environmental behaviors of EE2 in the northern Taihu Lake.

## 2. Materials and Methods

### 2.1. Sample Collection

Three surface sediment samples were collected from northern Taihu Lake in May 2013. Sampling site 1 (S1, 120°24.297′E, 31°26.593′N), S2 (120°09.012′E, 31°29.567′N), and S3 (120°03.010′E, 31°27.717′N) are located in Wangyu River estuary of Gonghu Bay, Zhihu Harbor estuary of Meiliang Bay, and Caoqiao River estuary of Zhushan Bay, respectively. The sediment samples were collected from approximately 0–20 cm under the bottom surface of the lake using Peterson dredge. The collected samples were freeze-dried and milled with agate mortar. Then they were sieved with 0.2 mm nylon sieve. All the treated samples were stored at −4°C until further analysis.

### 2.2. Materials and Instruments

Two estrogens, EE2 and BPA, were purchased from Aldrich (Milwaukee, WI, USA). Acetonitrile and methanol were obtained from Merk (Darmstadt, Germany). Hydrochloric acid (HCl) and sodium hydroxide (NaOH) were gained from Nanjing Chemical Reagent Company (Nanjing, China). Ultrapure water was obtained from Mill-Q water (MF-A10, Millipore, USA). Rotary mixer was obtained from Weicheng Experimental Equipment Factory (Jintan, China). The pH meter PHS-3C was obtained from Shanghai Precision Scientific Instrument Co., (Shanghai, China).

### 2.3. Adsorption Kinetics of EE2

Sediment samples from S3 were applied for adsorption kinetics experiment to determine adsorption equilibrium time of EE2. Sediment sample (4 g) and electrolyte solution (200 mL) were added into 250 mL conical flask. Background electrolyte solutions were 1 M CaCl_2_ and 200 mg/L NaN_3_ (antibacterial agents). EE2 stock solution in methanol was added into Mud suspension, and the initial concentration of EE2 in water was 2 mg/L. In order to avoid the eutectic effect of adsorption process, the final volume ratio of methanol was controlled less than 0.1%. The flask was mixed in the rotary mixer at room temperature. After that, the samples obtained from different times were centrifugated at 5000 rpm for 30 min. EE2 concentrations in supernatant were measured by high performance liquid chromatography (HPLC), and the EE2 concentration in the sediment was calculated according to subtraction. Two parallel samples were performed at each time, and soilless control experiments were also carried out.

### 2.4. Adsorption Isotherms of EE2

Sediment sample (0.5 g) and electrolyte solution (25 mL) were added into 40 mL brown glass bottle. Background electrolyte solutions were the same to the adsorption kinetics. The EE2 stock solution was transferred into Mud suspension to form a series of concentrations: 0.5,  1, 2,  3,  4, and 5 mg/L. And the volume ratio of methanol was controlled less than 0.1%. The solutions were mixed in the rotary mixer at room temperature. According to the results of adsorption kinetics experiments, 24 h was taken as the sorption equilibrium time. After adsorption equilibrium, the Mud mixture was centrifugated at 5000 rpm for 30 min. The EE2 concentrations in the supernatant were measured by HPLC, and the EE2 concentrations in the sediment phase were calculated by subtraction.

### 2.5. Influences of pH and BPA of EE2 Adsorption

The influences of pH on EE2 adsorption were investigated. pH values were adjusted by HCl and NaOH solution. During adsorption experiment, initial EE2 concentration was set to 2 mg/L. Other procedures were the same with the adsorption kinetic and isotherm test. Besides pH, the competition of BPA was also analyzed. Three BPA concentrations (1, 10, and 40 mg/L) were applied.

### 2.6. HPLC Analysis

The HPLC analysis was performed using Waters e2695 consisting of Diode array detector (PDA) of Waters 2998 with the detection wavelength of 200–280 nm. The column temperature and injection volume were 30°C and 10 *μ*L, respectively. A mixture of acetonitrile (A) and pure water (B) were used as the mobile phases at the constant flow rate of 1 mL/min. When there was only EE2 in the solutions, the mobile phase (60% A) was maintained for 4 min. However, when EE2 and BPA coexisted, the steps of gradient elution were as follows: maintaining the acetonitrile (A) concentration at 40% (initial condition) for 3 min, then increasing to 60% for 3.5 min, and finally holding 5 min. The relative standard deviation (RSD) determined by triplicate analyses of samples spiked at 300 *μ*g/L EE2 was 4.2%, indicating satisfactory precision. The limit of detection (LOD) calculated at a signal to noise ratio (S/N) of 3 was 95 *μ*g/L.

## 3. Results and Discussion

### 3.1. Adsorption Kinetics of EE2

**Figure 1:**
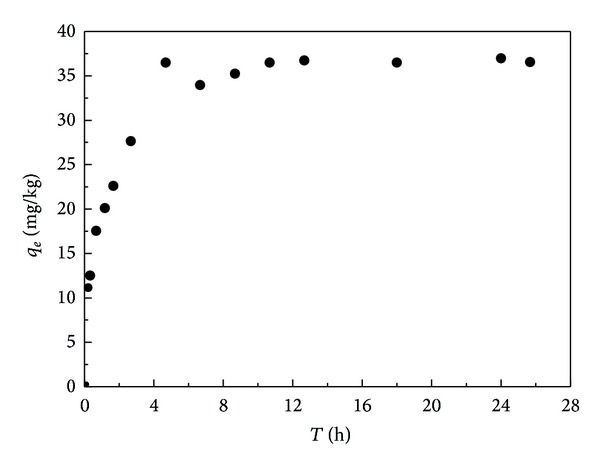
Adsorption kinetic curve of EE2 in the S3 sediment.

Adsorption kinetics of EE2 in the sediment (S3) is shown in Figure 1. The adsorption process contained an increase phase and an equilibrium phase, which was consistent with reports from Li et al. [6]. The adsorption rate increased very quickly within 4 h. For example, the adsorption amounts reached 1/3 and 1/2 of the adsorption equilibrium quantity at 10 and 40 min, respectively. The rapid adsorption stage might be due to the roles of organic matter in the sediment. The adsorption amount increases very slowly after 7 h. This stage was considered as irreversible diffusion or impaction into the micropores [6, 7]. The soilless control experiments showed that the loss of EE2 due to volatilization, degradation, and adsorption on the bottle wall during the test was negligible. Based on above results, 24 h was selected as the adsorption equilibrium time.

### 3.2. Adsorption Isotherms of EE2

**Figure 2:**
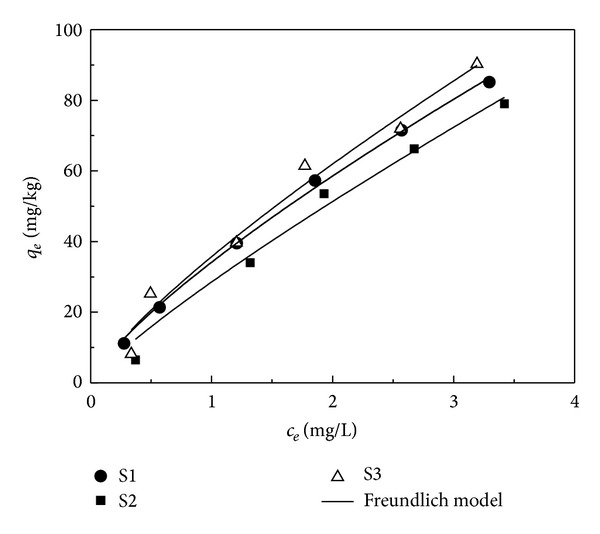
Adsorption isotherms of EE2 in the sediment.

Adsorption isotherms of EE2 in different sediment samples are shown in Figure 2. The Freundlich model (1) was used to fit the sorption isotherms of EE2:

(1) $$\begin{array}{c} q_{e}=K_{F}c_{e}^{1/n}, \end{array}$$

where *q*_*e*_ (mg/kg) is adsorption capacity of EE2 in the sediment, *K*_*F*_ (mg^1−1/*n*^/(L^1/*n*^ · kg)) is Freundlich sorption coefficient, and *c*_*e*_ (mg/L) is EE2 concentration in aqueous phase after adsorption equilibrium. 1/*n* is an empirical constant, which means the nonlinear degree of isotherms. When 1/*n* > 1, the sorption isotherm is concave upward, indicating that the adsorbed solutes change the surface structure of the adsorbents, thereby the absorption being further promoted. When 1/*n* < 1, the sorption isotherm is concave downward, which means that the adsorption occurs on the nonhomogeneous adsorbent surface or the dense organic matter, and the solutes occupy the highest energy point and the lower energy points successively. When 1/*n* = 1, it illustrates that the solutes distribute in the organic matter or adsorb on the hydrophilic minerals of the sediment. Fitting parameters obtained in this study are shown in Table 1.

As shown in Figure 2 and Table 1, the Freundlich model fitted well with the adsorption isotherms of EE2 in the sediments (*R* ^2^ > 0.96). 1/*n* was in the range of 0.7772–0.8463, indicating that the sorption of EE2 in the sediment is nonlinear adsorption. In order to explain the behaviors of nonlinear adsorption organics, distributed reactivity model (DRM) was proposed [8–10]. The model thought that organic matter is a kind of heterogeneity macromolecular polyelectrolyte on the composition and structure, which is made up of rubber phase (the region of soft carbon structure or amorphous organic matter) and glass phase (the region of rigid carbon structure or dense organic matter). Adsorption of hydrophobic organic compounds in the organic matter of rubber phase is mainly distribution effect-linear absorption, while in the glass phase it contained distribution function and adsorption function occurring on its internal microporous surface-nonlinear adsorption. When the organic carbon content in sediment is higher than 0.1%, the adsorption of organic compounds occurs mainly in the organic matter section. Our results indicated that the adsorption of EE2 in the sediment might be decided by many factors, and adsorption isotherms showed the adsorption process of BPA was nonlinear.

Based on the Freundlich constant *K*_*F*_ in the three sediments, adsorption ability of the three sediments was in the order S3 > S1 > S2. The adsorption of EE2 in the sediment was closely related to the properties of sediment. Comparing with SOM contents in different sediments, the order of adsorption capacity was consistent with the order of SOM contents, indicating that high SOM content could increase the adsorption capacity for EE2. These results are in accordance with the previous reports [11, 12].

### 3.3. pH Dependence of Sorption

**Figure 3:**
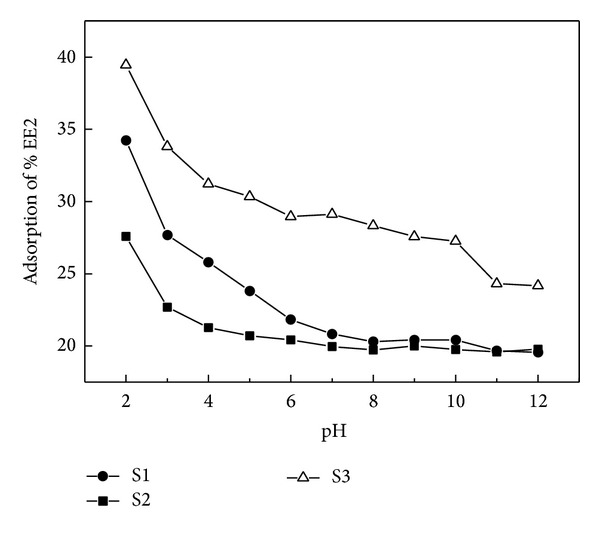
Effects of pH on the EE2 adsorption.

Influences of pH on EE2 adsorption are shown in Figure 3. With the increase of pH, the adsorption capacities of three sediments for EE2 decreased. The adsorption amounts of EE2 in S1, S2, S3 in pH = 1 were 1.75, 1.39, and 1.63 times higher than those in pH = 12, respectively. There might be two major reasons for pH influences. One reason might be that EE2 is a weakly acidic organic compound; another is its acid dissociation constant (pKa ≈ 10). When pH is in the range of 2–12, EE2 exists in two forms: molecule and ion. With the increase of pH value, more EE2 is ionized, especially the anionic form of EE2. Then the electrostatic repulsion between the anionic form of EE2 and sediments with negative charge became more obvious [13, 14]. In addition, with the increasing of pH, the organic matters in the sediments would be dissolved, which made the hydrophilicity of sediment be stronger; thus the adsorption amount of EE2 reduced [15]. Similar research performed by Li et al. [15] found that the adsorption amount of BPA in the sediment decreased with the increasing of pH value as well.

### 3.4. Influence of BPA on EE2 Adsorption

In general, competitive adsorption occurs when two or more contaminants coexisted in the sediment-water system, which could reduce the adsorption capacity of sediment and improve desorption rate and toxicity of contaminants [16–18]. Thus, identification of competitive adsorption is very important for the risk assessment of target pollutant. In this study, we studied the competitive effects of BPA on EE2 adsorption in sediment. BPA and EE2 as important environmental estrogens have been widely researched. There are many different chemical properties between them, such as different chemical structures and hydrophobicity. The molecular diameter of BPA is 0.43 nm, which is less than the value of EE2 (0.6 nm) [19]. The water solubility of BPA is about 50 times higher than that of EE2.

**Figure 4:**
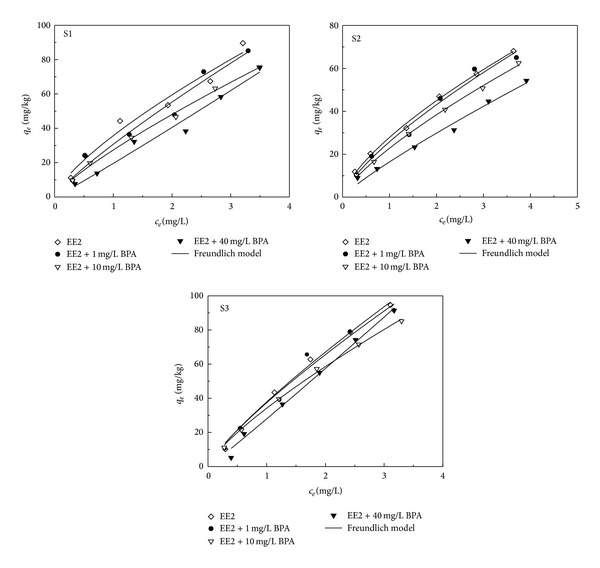
Effects of BPA on the adsorption isotherms of EE2 in different sediment samples. The EE2 concentration was 2 mg/L.

Influences of BPA on adsorption isotherms of EE2 in the three sediments are shown in Figure 4 and Table 2. When BPA existed, the Freundlich adsorption isotherm fitting curves could also describe EE2 adsorption well (*R* ^2^ > 0.95). With the increases of BPA concentrations, the *K*_*F*_ of adsorption isotherms decreased. These results suggested that increase of BPA concentrations could inhibit EE2 adsorption. However, with the increase of BPA concentration, the 1/*n* values increased, more close to 1, indicating that the adsorption isotherm became more linear, and the energy distribution after competitive adsorption turned more uniform.

When the concentrations of BPA and EE2 were similar, the inhibition effects of BAP on EE2 adsorption were not obvious, which might be due to the different hydrophobicity of the two substances. As described above, EE2 adsorption mainly occurred in the SOM of sediment. The inner or surface of hard carbon of SOM owned hydrophobic adsorption sites. The substance with higher hydrophobicity is more easily adsorbed in the SOM [20, 21]. Thus, no significant influences in EE2 adsorption were found under low BPA concentration. However, when BPA concentration reached 40 mg/L, the competitive adsorption between EE2 and BPA not only depended on differences of hydrophobicity but also was influenced by other factors. Braida et al. [22] suggested that the surface of SOM was mainly composed of micropores with the maximum pore diameter of 0.3–0.5 nm. Smaller diameter molecule BPA might have more adsorption sites than EE2. In addition, BPA has one more benzene ring with a hydroxyl group than EE2. The benzene ring can form *π*-*π* bond with the hard carbon part of SOM; therefore, the *π*-*π* bond formed between the hard carbon of SOM and BPA is stronger than that of EE2 [19]. The above reasons resulted in the higher adsorption capacity of BPA and significant inhibition to the adsorption of EE2.

The chemicals with different properties can complementarily occupy different sites of adsorbents [20]. At low concentrations, BPA and EE2 are adsorbed on high energy points firstly. But different qualitative substances present different affinity to different points. For example, BPA has stronger affinity to the groove area of the sediment, but EE2 is easy to be adsorbed in the hydrophobic surface of the sediment. These might be the reasons that the competitive adsorption made the energy distribution more uniform and the adsorption isotherms become more linear.

## 4. Conclusions

This study firstly analyzed the adsorption behaviors of EE2 in sediment-water system in northern Taihu Lake. Freundlich model could simulate the adsorption isotherms of EE2 in the sediments (*R* ^2^ > 0.96). The properties of sediment, especially the SOM content, influenced EE2 adsorption. In addition, pH value also influenced the EE2 adsorption. Low pH values could increase the adsorption of EE2. When high level BPA coexisted, the adsorption capacity of sediment for EE2 decreased. Our results provide primary information of EE2 adsorption in sediment-water system in Taihu Lake, which is useful for the environmental risk assessment and management of EE2 in studied area.

## Figures and Tables

**Table 1:** Fitting parameters of Freundlich model.

Site	SOM	*K*_*F*_	1/*n*	*R* ^2^
S1	30.8	34.16	0.7772	0.9974
S2	28.2	28.56	0.8463	0.9778
S3	32.5	35.72	0.7943	0.9699

**Table 2:** Fitting parameters of Freundlich equation for EE2 adsorbed in the three sediments under different concentrations of BPA.

Site	Freundlich model	BPA (mg/L)			
		0	1	10	40
S1	*K*_*F*_	35.69	33.68	27.36	19.55
	1/*n*	0.7357	0.8485	0.8718	1.0498
	*R* ^2^	0.9620	0.9556	0.9951	0.9677

S2	*K*_*F*_	27.83	25.78	22.78	16.40
	1/*n*	0.6910	0.7392	0.7517	0.8621
	*R* ^2^	0.9961	0.9756	0.9978	0.9830

S3	*K*_*F*_	36.87	36.35	34.16	27.93
	1/*n*	0.8236	0.8114	0.7777	1.0400
	*R* ^2^	0.9932	0.9704	0.9974	0.9909
